# Characterization of NLRP12 during the *In Vivo* Host Immune Response to *Klebsiella pneumoniae* and *Mycobacterium tuberculosis*

**DOI:** 10.1371/journal.pone.0060842

**Published:** 2013-04-05

**Authors:** Irving C. Allen, Erin McElvania-TeKippe, Justin E. Wilson, John D. Lich, Janelle C. Arthur, Jonathan T. Sullivan, Miriam Braunstein, Jenny P. Y. Ting

**Affiliations:** 1 Lineberger Comprehensive Cancer Center, The University of North Carolina at Chapel Hill, Chapel Hill, North Carolina, United States of America; 2 Department of Biomedical Sciences and Pathobiology, Virginia Tech, Virginia-Maryland Regional College of Veterinary Medicine, Blacksburg, Virginia, United States of America; 3 Department of Microbiology and Immunology, The University of North Carolina at Chapel Hill, Chapel Hill, North Carolina, United States of America; Louisiana State University, United States of America

## Abstract

The majority of nucleotide binding domain leucine rich repeats-containing (NLR) family members has yet to be functionally characterized. Of the described NLRs, most are considered to be proinflammatory and facilitate IL-1β production. However, a newly defined sub-group of NLRs that function as negative regulators of inflammation have been identified based on their abilities to attenuate NF-κB signaling. NLRP12 (Monarch-1) is a prototypical member of this sub-group that negatively regulates both canonical and noncanonical NF-κB signaling in biochemical assays and in colitis and colon cancer models. The role of NLRP12 in infectious diseases has not been extensively studied. Here, we characterized the innate immune response of *Nlrp12^−/−^* mice following airway exposure to LPS, *Klebsiella pneumoniae* and *Mycobacterium tuberculosis*. In response to *E. coli* LPS, *Nlrp12^−/−^* mice showed a slight decrease in IL-1β and increase in IL-6 production, but these levels were not statistically significant. During *K. pneumoniae* infection, we observed subtle differences in cytokine levels and significantly reduced numbers of monocytes and lymphocytes in *Nlrp12^−/−^* mice. However, the physiological relevance of these findings is unclear as no overt differences in the development of lung disease were observed in the *Nlrp12^−/−^* mice. Likewise, *Nlrp12^−/−^* mice demonstrated pathologies similar to those observed in the wild type mice following *M. tuberculosis* infection. Together, these data suggest that NLRP12 does not significantly contribute to the *in vivo* host innate immune response to LPS stimulation, *Klebsiella pneumonia* infection or *Mycobacterium tuberculosis*.

## Introduction

Initiation and termination of the host innate immune response to pathogenic bacteria infection is tightly regulated. The host response to respiratory bacteria is initially driven through the stimulation of TLRs. TLR activation signals through the NF-κB pathway to up-regulate the transcription of proinflammatory cytokines, including IL-1β, TNFα, and IL-6, which are essential for the recruitment of leukocytes to the lung and clearance of the infection. Mice deficient in these three proinflammatory cytokines have demonstrated significant increases in morbidity and mortality during respiratory infections with *Klebsiella pneumoniae* and *Mycobacterium tuberculosis* [1], [2].

While TLR recognition occurs on the plasma membrane and in endosomes, the nucleotide binding domain leucine rich repeats-containing family of proteins (NLRs) are a second family of pathogen recognition receptors (PRRs) that sense intracellular danger associated molecular patterns (DAMPs) and pathogen associated molecular patterns (PAMPs) in the cell cytoplasm [3], [4], [5], [6]. Thus far, over 23 distinct NLRs have been identified; however, the biological functions of the majority of these proteins have yet to be elucidated. The bulk of NLR publications have sought to characterize a sub-group of NLRs that function in the formation of a multi-protein complex termed the inflammasome, which includes a specific NLR, the adaptor protein PYCARD and Caspase-1. An inflammasome forms following the activation of a particular NLR by a specific danger signal, which ultimately results in the activation of caspase-1 and the subsequent post-translational cleavage of pro-IL-1β and pro-IL-18 into their active forms.

Unlike the majority of characterized proinflammatory NLRs, recent studies have identified a novel sub-group of NLRs that have the ability to attenuate inflammation [7], [8], [9], [10], [11], [12], [13]. The exact mechanism underlying this negative regulation is an area of active investigation; however, it appears that most of these NLRs function by inhibiting components of NF-κB signaling. The NLR, NLRP12 (formally known as MONARCH-1 or PYPAF7), is a prototypical member of this sub-group that functions as a negative regulator of the immune system through the attenuation of non-canonical NF-κB signaling and p52-dependent chemokine expression [12], [14]. This suppression is facilitated by the association of NLRP12 with the proteasome and the subsequent proteasome-dependent degradation of NF-κB inducing kinase (NIK) [12]. The interaction of NLRP12 with components of the NF-κB pathway ultimately results in the attenuated production of proinflammatory cytokines following PAMP stimulation and bacterial infections. *In vivo*, NLRP12 has been shown to dramatically influence the development of contact hypersensitivity, gastrointestinal inflammation and tumorigenesis [14], [15], [16].

Bacterial mediated infectious lung diseases are an important worldwide cause of morbidity and mortality. The gram negative bacterium *Klebsiella pneumoniae* is a leading cause of community- and hospital-acquired respiratory infection. The growing prevalence of antibiotic resistant strains constitutes a serious public health concern [17]. Pathogenic *K. pneumoniae* is capable of inducing severe bacterial pneumonia that is characterized by extensive lung inflammation, hemorrhage and necrotic lesion formation in the lungs, which can often progress to bacteremia and sepsis [18]. Similar to *K. pneumoniae*, the incidence of *Mycobacterium tuberculosis* (*Mtb*) is also a global threat based on the increasing prevalence of antibiotic resistant *Mtb* [19], [20]. One third of the world’s population is infected with *Mtb* and tuberculosis is associated with 1.4 million deaths per year [20]. The majority of patients infected with *Mtb* develop a latent tuberculosis infection. Individuals that are latently infected with *Mtb* will maintain a significant lifetime risk of disease reactivation despite being asymptomatic [20]. Disease reactivation is typically associated with some level of immune system compromise, such as HIV co-infection, or as a consequence of immunosuppressive drug therapy [21]. Thus, a deeper understanding of the underlying immune mechanisms that regulate active and latent *Mtb* infection is of immense clinical importance.

The innate immune response to *K. pneumoniae* and *Mtb* is influenced by NLR family members. Components of the NLRP3 inflammasome directly mediate the *ex vivo* and *in vitro* production of pro-inflammatory cytokines in response to *K. pneumoniae* infection in primary macrophages and monocytic cell lines [1]. Likewise, NLRP3 inflammasome components are essential for host defense against *K. pneumoniae in vivo* through the regulation of IL-1β production and necrosis in the lungs [1]. Components of the NLRP3 inflammasome are also essential for the production of active IL-1β in cultured human monocytes and primary mouse macrophages infected with *Mtb*. In addition, the NLR inflammasome adaptor protein PYCARD (ASC) exerts a novel NLRP3-independent role in granuloma formation and maintenance during *in vivo Mtb* infection [22]. Other NLRs, including NLRP12, have also been evaluated in the host immune response to gram negative bacteria and *Mtb in vitro*. Studies in the THP-1 human monocytic cell line found that *Nlrp12* is highly expressed at baseline levels, and expression is down-regulated following stimulation with TNF-α, IFN-γ, or *Mtb* [10]. Downregulation of NLRP12 was suggested to be an important step in allowing the host to mount an effective inflammatory response [10]. Functionally, shRNA knockdown of *NLRP12* results in increased levels of pro-inflammatory cytokines following LPS or *Mtb* challenge [10]. However, these studies were performed *in vitro* with human cell lines that express high basal levels of *NLRP12*; thus, validation in a more physiologically relevant setting is necessary. While several studies have assessed the contribution of proinflammatory NLRs in host defense, few studies have explored the *in vivo* role of the NLR family members that exert negative regulatory activities in host defense against pathogenic bacteria.

The identification and *in vivo* characterization of proteins that function as negative regulators of inflammation is an area of intense scientific and clinical importance for the development of clinical therapies. Previous studies have shown that NLRP12 functions as a negative regulator of inflammatory pathways *in vitro*; however, the role of NLRP12 in regulating host-pathogen interactions *in vivo* is not well defined. Here, we tested the hypothesis that mice lacking *Nlrp12* would demonstrate increased morbidity, mortality and pathology following *in vivo K. pneumoniae* or *Mtb* infection. Based on the previous *in vitro* findings, we expected to observe accelerated disease progression, lung inflammation and cytokine production associated with an overzealous immune response in the *Nlrp12^−/−^* mice following *K. pneumonia* or *Mtb* bacterial infection.

## Materials and Methods

### Experimental Animals

All studies were conducted under the approval of the Institutional Care and Use Committee (IACUC) for The University of North Carolina at Chapel Hill and in accordance with the National Institutes of Health Guide for the Care and Use of Laboratory Animals. *Nlrp12^−/−^* animals were kindly provided by Millennium Inc. and previously described [15]. All mice were maintained under specific pathogen free conditions and all experiments were performed with 6–12 week old age- and sex- matched mice. All mice were backcrossed onto C57Bl/6J for a minimum of 12 generations. *Nlrp12^−/−^* mice were compared to both purchased C57Bl/6J mice and littermate control mice from heterozygous breeding. All *in vivo Mtb* work was conducted under approved BSL-3/ABSL-3 protocols and conditions.

### Evaluation of Cytokine Levels in Primary Dendritic Cells

Wild type and *Nlrp12^−/−^* dendritic cells were isolated and cultured as previously described [14]. Once matured, the dendritic cells were stimulated with either 50 ng/ml of *E. coli* 0111:B4 LPS (Invivogen), 50 ng/ml of *Klebsiella pneumoniae* LPS (Sigma; source strain is ATCC 15380) or 50 µg/ml of trehalose-6,6-dibehenate (TDB), which is a synthetic analog of trehalose-6,6-dimycolate from *Mycobacterium tuberculosis* (Invivogen). Cell culture supernatants were collected following 4 hours of stimulation and TNF-α, IL-6 and IL-1β levels were assessed using ELISA (BD Biosciences). To evaluate IL-1β maturation and release, cells were treated with 2 mM ATP for 30 minutes prior to harvesting the supernatants. Pro- and cleaved IL-1β was detected by western blot in dendritic cell lysate and supernatant following 24 hours of LPS stimulation. Following the supernatant harvest, the remaining cells were lysed in 1% RIPA buffer. Immunoblots were probed with goat anti-mouse IL-1β primary antibody (R&D Biosystems), followed by rabbit anti-mouse HRP secondary antibody (Santa Cruz). Bands were visualized by Super Signal Chemiluminescence (Pierce).

### Induction and Assessment of Acute Airway Inflammation

To assess lipopolysaccharide (LPS) induced acute airway inflammation, isoflurane inhalation was used to anesthetize the mice, and LPS (Sigma) isolated from *Escherichia coli* (sterile serotype 0111:B4) was instilled via intratracheal (i.t.) administration as previously described [23]. Control animals received an i.t. dose of saline. Mice were euthanized and airway inflammation was assessed 48 hours post LPS exposure.

Bacterial mediated acute airway inflammation was induced by *K. pneumoniae* lung challenge. *K. pneumoniae* 43816 (serotype 2) was obtained from the ATCC and cultured under suppliers recommended conditions. Prior to each experiment, bacteria density was estimated by measuring the absorbance at 600 nm and actual colony forming units (CFUs) were determined by plating an aliquot on LB agar plates. Mice were challenged via i.t. instillation with either 7.4×10^4^ CFUs of *K. pneumoniae* in 50 µl of PBS or PBS alone (mock), as previously described [1]. To assess *in vivo* bacteria burden, mice were euthanized via i.p. injection with 2,2,2 tribromoethanol (avertin), bronchoalveolar lavage fluid (BALF) was collected as described below, and the cell free fluid was serially diluted and plated on LB agar plates. Additionally, the liver was removed, wet weight assessed, homogenized in 500 µl of HBSS with a Tissue Master 125 (Omni International) and centrifuged. The resulting supernatants were plated on LB agar plates.

### Whole Body Aerosol Infection with *Mycobacterium tuberculosis*

*Mycobacterium tuberculosis* H37Rv strain was obtained from the American Type Culture Collection (ATCC). Bacteria were grown to log phase in Middlebrook 7H9 broth (Difco) with 0.2% glycerol, 1× albumin dextrose saline (ADS), and 0.05% Tween 80. Inoculum was assessed by plating infection media on Middlebrook 7H10 agar plates supplemented with glycerol and ADS. CFUs were counted after 21 days of incubation.

For *in vivo* airway infection, *Nlrp12^−/−^* and wild type mice were infected via aerosol as previously described [24]. Mice received 250–350 CFUs per lung, which was determined by sacrificing a subset of mice 1 day post infection as previously described [24]. Bacterial organ burden was quantified by plating serial dilutions of lung, liver, and spleen homogenates on 7H10 agar containing cycloheximide (1 µg/ml) and carbenicillin (50 µg/ml) to minimize contamination. CFUs were counted after 21 days of incubation and again after 35 days of incubation. All animal infections and organ harvests were carried out under BSL3/ABSL3 conditions.

### Evaluation of Airway Inflammation

Upon completion of the LPS and *K. pneumoniae* models, mice were euthanized and serum was collected from animals by cardiac puncture. Bronchoalveolar lavage fluid (BALF) was collected to evaluate leukocytes and cytokine levels. The lungs were lavaged three times with 1 ml of HBSS. Following centrifugation, the cytokine levels in the cell free supernatants were evaluated by ELISA (BD Biosciences). Total BALF cellularity was determined from the resuspended cell pellet and evaluated using a hemocytometer. The composition of the BALF was determined by evaluating the morphology of ≥200 cells per sample following differential staining (Diff-Quik, Dade Behring) of cells that were cytospun onto slides.

At specific time points post-*Mtb* infection, mice were euthanized and the liver, spleen and right lung lobes were collected and homogenized for bacteria CFU analysis and cytokine profiles. Tissue homogenates were serial diluted and plated to determine CFUs. The remaining lung homogenates were centrifuged to remove cellular debris and the supernatant was double filter sterilized for cytokine analysis by ELISA.

For LPS and *K. pneumoniae* histopathology, whole lungs were fixed by inflation to 20-cm pressure and immersion in 10% buffered formalin. To evaluate *Mtb* histopathology, the left lung lobe was removed, manually inflated and immersed in buffered formalin. Inflammation was evaluated in sections (5 µm) of the left lung lobe, following hematoxylin and eosin (H&E) staining. Paraffin embedded sections were set and cut to reveal the maximum longitudinal visualization of the intrapulmonary main axial airway. Histopathology was evaluated and scored by blinded reviewers on a scale of 0 (absent) to 3 (severe), as previously described [1], [23], [25]. The parameters assessed included overall leukocyte infiltrations, perivascular and peribroncheolar cuffing, airway epithelial cell injury, extravasation, and the estimated percent of lung area involved with inflammation. Each individual parameter was scored and averaged to generate the histology score. Additional parameters were used to evaluate the granulomas in the *Mtb* histopathology as previously described [22]. Briefly, granuloma frequency was measured by counting the number of granulomas present in the total lung section, and granuloma size was evaluated using Image J software after the granuloma borders were defined. Ziehl-Neelsen (ZN) staining was utilized to evaluate acid fast bacterial localization within the lung and granuloma as previously described [22].

### Statistical Analysis

We utilized GraphPad Prism 5 Statistical software to conduct Analysis Of Variance (ANOVA) followed by either Tukey-Kramer HSD or Newman-Keuls post-test to evaluate statistical significance for multiple comparisons. Single data point comparisons were evaluated by the Student’s two-tailed t-test. All data are presented as the mean +/− the standard error of the mean (SEM), and in all cases a p-value of less than 0.05 was considered statistically significant.

## Results

### NLRP12 Attenuates the Release of TNFα and IL-6 in Bone Marrow Derived Dendritic Cells, but does not Contribute to IL-1β Maturation or Release

**Figure 1:**
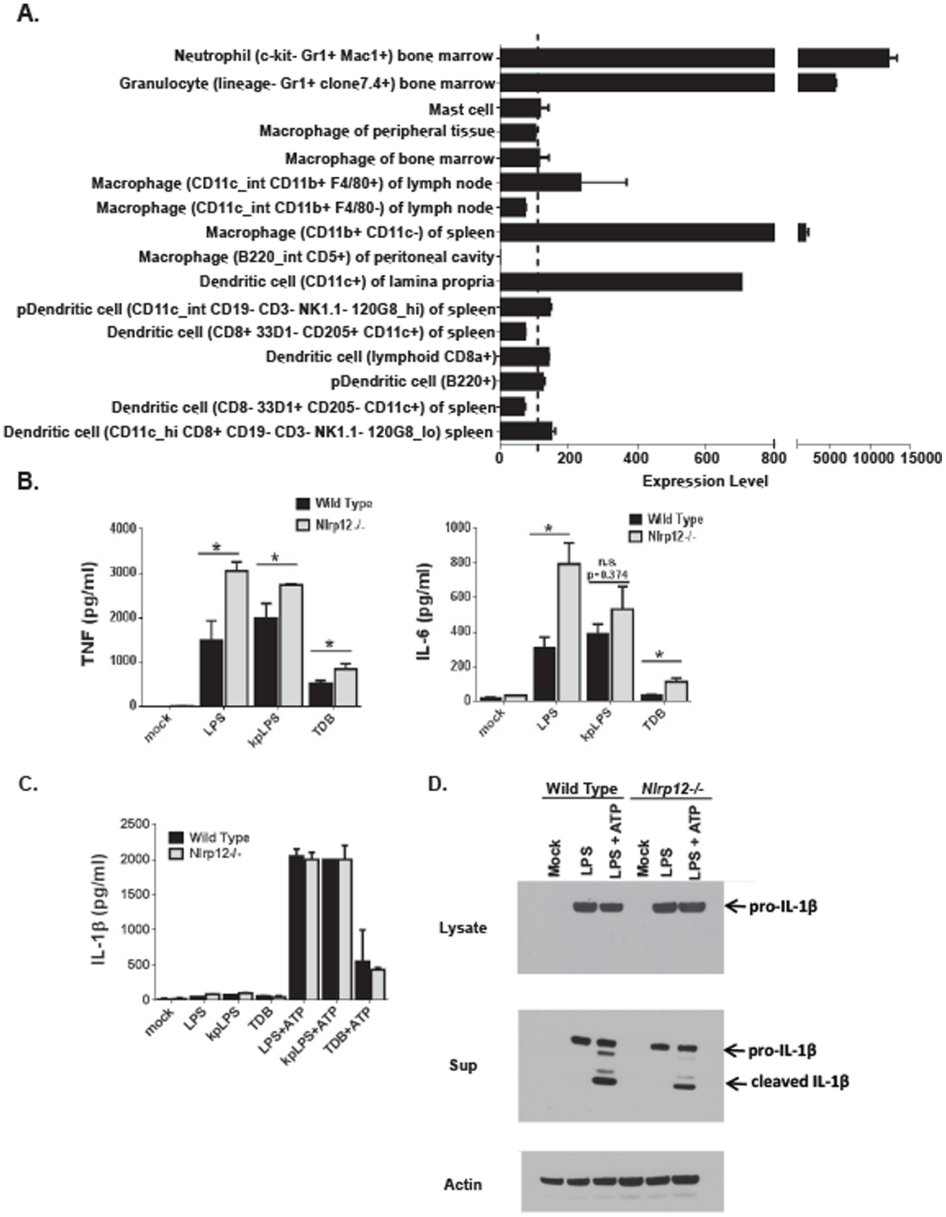
Dendritic cell stimulation with PAMPs associated with *E. coli*, *K. pneumoniae* and *M. tuberculosis*. **A)** *Nlrp12* expression in relevant immune system cell types was compiled using a publically accessible microarray meta-analysis search engine (Nextbio website. Available: http://www.nextbio.com/b/search/ba.nb. Accessed 2013 March 11). **B)** Primary bone marrow derived dendritic cells were isolated, cultured and stimulated with either *E. coli* LPS (LPS)(50 ng/ml), *K. pneumoniae* LPS (kpLPS)(50 ng/ml) or trehalose-6,6-dibehenate (TDB; a synthetic analog of TDM from *M. tuberculosis*)(50 µg/ml). The levels of TNFα and IL-6 were evaluated in the supernatants following 4 hours of stimulation. Experiments were repeated at least 3 separate times with dendritic cells harvested from 3 individual mice for each genotype and treatment in each replicate. **C)** IL-1β was evaluated in the supernatants 4 hours after stimulation, following an additional treatment with 2 mM ATP 30 minutes prior to harvesting the supernatants. Experiments were repeated at least 3 separate times with dendritic cells harvested from 3 individual mice for each genotype and treatment in each replicate. **D)** Pro- and cleaved IL-1β was detected by western blot in dendritic cell lysate and supernatant following LPS stimulation. Experiments were repeated at least 3 separate times with dendritic cells harvested and pooled from 3 individual mice from each genotype and treatment in each replicate.

Previous studies characterizing NLRP12 in human monocyte cell lines revealed that NLRP12 functions as a negative regulator of NF-κB signaling following stimulation with TLR agonist, TNFα and infection with *M. tuberculosis* [10], [12]. To expand upon these previous findings, we sought to evaluate the contribution of NLRP12 in mediating the host immune response in mice. Initially, we utilized bioinformatics to evaluate *Nlrp12* expression patterns in specific cell subpopulations. *Nlrp12* expression in mouse cells associated with the host immune response was compiled using a publically accessible microarray meta-analysis search engine (Nextbio website. Available: http://www.nextbio.com/b/search/ba.nb. Accessed 2013 March 11), as previously described [14]. This analysis revealed highly variable levels in the expression of *Nlrp12* in select cell populations relevant to the host immune response. *Nlrp12* was found to be highly expressed in neutrophils and consistently expressed in dendritic cell populations (**Figure 1A**). However, macrophage *Nlrp12* expression was highly variable. For example, cell populations from the spleen expressed high levels of *Nlrp12,* whereas populations from the peritoneal cavity did not have any detectable levels (**Figure 1A**). To evaluate cytokine production in response to pathogen stimulation, dendritic cells were isolated from the bone marrow, cultured and matured as previously described [14]. These primary cells were stimulated with pathogen associated molecular patterns (PAMPs) associated with *E. coli*, *K. pneumoniae* and *M. tuberculosis* infection. TNFα levels were significantly increased in *Nlrp12^−/−^* dendritic cell cultures following stimulation with *E. coli* derived LPS, *K. pneumoniae* derived LPS and the *M. tuberculosis* PAMP TDB (**Figure 1B**). Similarly, IL-6 levels were also significantly increased in *Nlrp12^−/−^* isolated cells stimulated with *E. coli* LPS and TDP (**Figure 1B**). Together, these data are consistent with previously published findings that suggest NLRP12 functions as a negative regulator of inflammatory signaling [10], [12], [14].

NLRP12 has been suggested to be capable of inflammasome formation and facilitate IL-1β maturation [26], [27]. Thus, in addition to evaluating TNFα and IL-6, we also evaluated IL-1β production and maturation using ELISA and western blot. We detected a significant increase in IL-1β release into the supernatant by ELISA following stimulation with all three PAMPs and ATP, in both *Nlrp12^−/−^* and wild type cells (**Figure 1C**). However, no significant differences were detected in IL-1β levels between the wild type and *Nlrp12^−/−^* cells (**Figure 1C**). Moreover, western blot analysis revealed that a significant amount of pro-IL-1β was present in the lysate from both wild type and *Nlrp12^−/−^* cells treated with LPS (**Figure 1D**). Likewise, both pro- and cleaved-IL-1β was consistently detected in the supernatant from wild type and *Nlrp12^−/−^* dendritic cells. Thus, these findings indicate that NLRP12 does not function in IL-1β maturation in mouse bone marrow derived dendritic cells following stimulation with the PAMPs tested. These data are consistent with other published findings that failed to support a role for NLRP12 in inflammasome formation under similar experimental conditions as those tested here [15].

### NLRP12 does not Play a Role in LPS Mediated Acute Lung Inflammation

**Figure 2:**
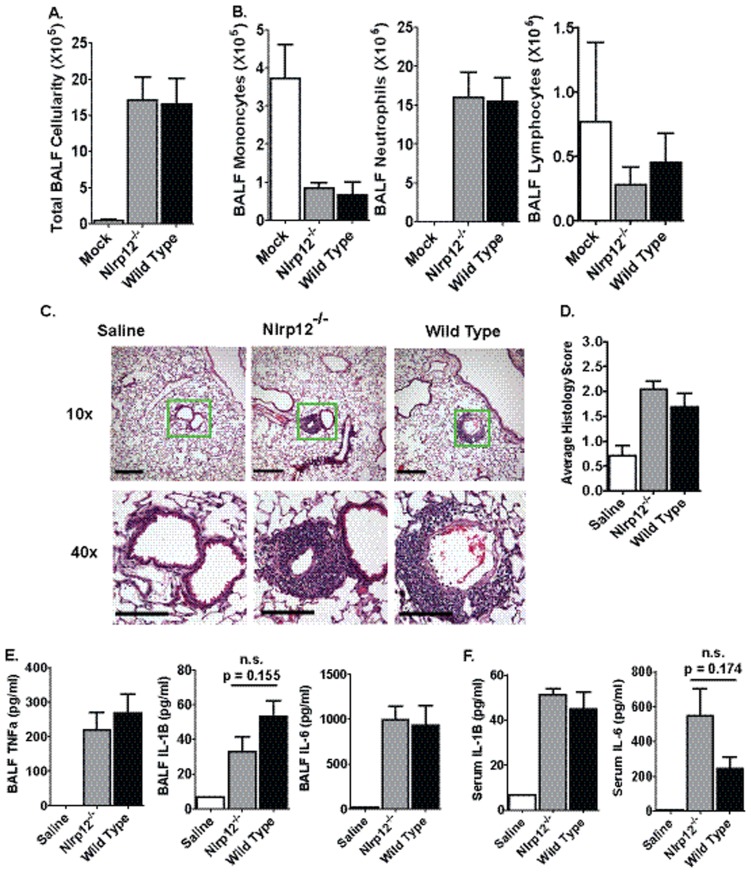
LPS mediated acute airway inflammation in *Nlrp12^−/−^* mice. Mice were challenged with 1 mg/kg of *E. coli* LPS (serotype 0111:B4) i.t. and airway inflammation was assessed 48 hours post-challenge. **A)** LPS induced a significant increase in BALF cellularity. **B)** No significant differences were detected in the cellular composition of the BALF between *Nlrp12^−/−^* and wild type mice. **C)** Airway challenge with LPS resulted in a significant influx of neutrophils to the airway. Histopathology revealed a significant amount of perivascular and peribroncholar cuffing and some slight alveolar occlusion following lung LPS exposure. The magnification bar at 10× = 10 µM and 40× = 5 µM. **D)** Histopathology analysis and histology scoring revealed no significant differences between the *Nlrp12^−/−^* and wild type mice. **E–F)** LPS induced a significant increase in local (BALF) and systemic (serum) levels of proinflammatory cytokines. No significant differences were observed between *Nlrp12^−/−^* and wild type mice. Saline, n = 3; LPS-treated *Nlrp12^−/−^*, n = 6; LPS-treated Wild Type, n = 6. Experiments were repeated 3 separate times with the same numbers of mice in each replicate.

Lipopolysaccharide (LPS) is a component of the cell wall of gram-negative bacteria and is ubiquitous in the environment. Inhalation of LPS has been shown to exacerbate airway reactivity in human asthmatics and activates the host innate immune system by acting as a PAMP to stimulate TLR4 receptors [28]. NLRP12 has previously been shown, *in vitro*, to down-regulate LPS induced NF-κB activation in human monocytes. This occurs through a mechanism that involves the inhibition of TLR/IL-1 receptor signaling proteins (MyD88, IRAK-1 and TRAF6) and the hyperphosphorylation of IRAK-1 [10]. These data suggest that NLRP12 may also function to down regulate the host innate immune response to LPS in a model of acute airway inflammation. *Nlrp12^−/−^* and wild type mice were challenged with 50 µg of LPS through intratracheal (i.t.) administration and harvested 16–20 hrs post-administration. The LPS challenge resulted in a significant increase in total bronchoalveolar lavage fluid (BALF) cellularity, which was consistent with a significant increase in airway neutrophilia following LPS treatment (**Figure 2A**). However, no significant differences were observed between *Nlrp12^−/−^* and wild type mice in either the total number or composition of the leukocyte populations present in the BALF (**Figure 2B**). This suggests that airway inflammation occurs independent of NLRP12 following LPS treatment.

Airway LPS exposure results in neutrophil infiltration of the lung parenchyma. Previous *ex vivo* data demonstrated that primary dendritic cells and neutrophils isolated from *Nlrp12^−/−^* mice displayed significantly attenuated cell migration in a model of skin hypersensitivity [15]. While BALF cellularity is a widely accepted and accurate surrogate marker for airway inflammation, it does not always fully reflect the extent of inflammation in the lung parenchyma. To further evaluate inflammation, lung histopathology was evaluated from the main bronchi of the left lobe and representative sections were examined (**Figure 2C**). Lung histopathology assessments revealed that LPS induced a significant increase in infiltrating neutrophils in the areas around the lung vasculature (perivascular), the lung parenchyma, and the large and small airways (peribronchiolar) (**Figure 2C**). However, no significant differences were observed in the lung histopathology between LPS challenged *Nlrp12^−/−^* and wild type mice (**Figure 2D**). Together, the BALF cellularity assessments and the histopathology findings show that NLRP12 does not play a significant role in LPS-induced acute lung inflammation.

Previous *in vitro* studies have demonstrated that NLRP12 functions as a negative regulator of TNFα signaling and the LPS-induced production of IL-6 and IL-1β in human monocyte cell lines [10]. However, the *in vitro* role of NLRP12 appears to be cell type and species specific. Studies utilizing *ex vivo* primary cells from *Nlrp12^−/−^* mice indicate that NLRP12 does not appear to influence the production of TNFα, IL-6 or IL-1β in mouse bone marrow derived dendritic cells [14], [15]. Thus, we sought to evaluate the *in vivo* contribution of NLRP12 in the local (BALF) and systemic (serum) cytokine response following airway administration of LPS (**Figure 2E–F**). We observed a significant increase in TNFα, IL-1β and IL-6 in the BALF following LPS challenge in both *Nlrp12^−/−^* and wild type mice (**Figure 2E**). Likewise, LPS challenge increased the serum levels of IL-1β and IL-6 (**Figure 2F**). TNFα was not detected in the serum following LPS challenge (data not shown). NLRP12 has been shown to associate with the inflammasome adaptor protein ASC/PYCARD, which suggests that NLRP12 may be capable of inflammasome formation under certain conditions [26], [27]. As seen in **Figure 2E and F**, BALF IL-1β levels were consistently attenuated and serum IL-6 levels were consistently increased in the *Nlrp12^−/−^* mice following LPS administration. However, these results did not achieve statistical significance. No other significant differences were observed in cytokine levels between the *Nlrp12^−/−^* and wild type mice (**Figure 2E–F**).

### Mice Lacking Nlrp12 Demonstrate Normal Immune Responses following *Klebsiella pneumoniae* Airway Infection

The use of bacterial LPS to induce acute lung inflammation is a well established and highly advantageous model that is commonly employed to assess the effects of Gram-negative bacteria. However, live bacterial pathogens activate the host innate immune system through a diverse range of PAMPs, effector proteins and antimicrobial peptides. Thus, one of the often cited limitations of LPS based models is that endotoxin exposure provides a limited view of the actual events that occur during a live bacteria infection in the lungs [29]. Several NLR proteins play integral roles in mediating the host innate immune response to a variety of lung pathogens. For example, NLRP3 is critical to the host immune response following airway infections with *Klebsiella pneumoniae* and *Chlamydia pneumoniae* [1], [30]. NLRC4 plays a vital role in regulating IL-1β and IL-18 production during lung mucosal defense following infections with *Pseudomonas aeruginosa* and *Burkholderia pseudomallei* [31], [32]. Likewise, the non-inflammasome forming NLR, NLRC2/NOD2, plays a role in neutrophil recruitment and bacterial killing during *E. coli*-mediated pneumonia in mice [33]. Thus, we next sought to determine if the host innate immune response is significantly altered in *Nlrp12^−/−^* mice during a live bacterial lung infection with *K. pneumoniae*.

**Figure 3:**
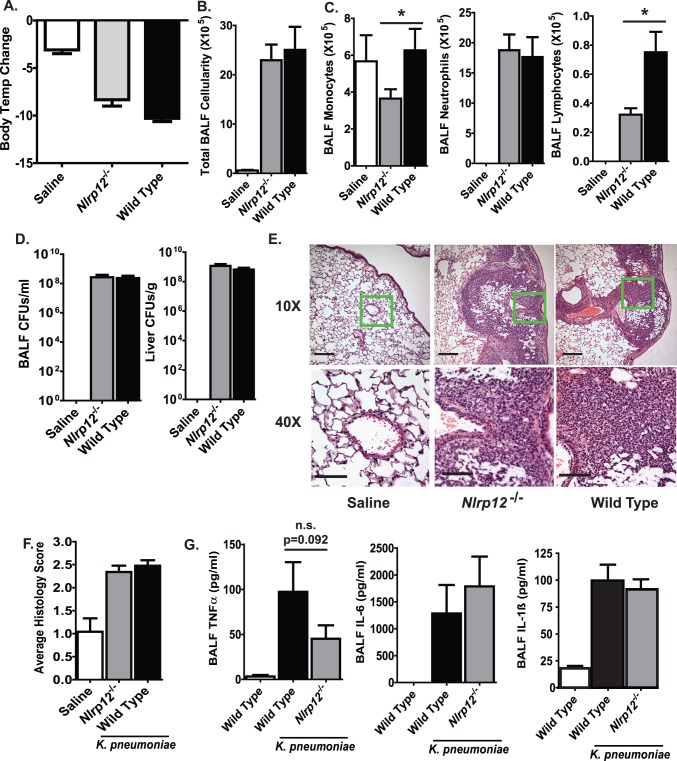
The host innate immune response to *Klebsiella pneumoniae* infection in *Nlrp12^−/−^* mice. A) Body temperature was monitored daily to assess morbidity. *K.pneumoniae* infection induced a significant decrease in body temperature in both *Nlrp12^−/−^* and wild type mice. **B)** The lungs were lavaged with HBSS and total BALF cellularity was determined. **C)** No significant differences in were observed in the BALF neutrophil populations between *Nlrp12^−/−^* and wild type mice. However, a significant decrease in the number of monocytes (p = 0.042) and lymphocytes (p = 0.032) were observed in the BALF from the *Nlrp12^−/−^* mice compared to the wild type. *P<0.05. **D)** No differences in bacterial burden were found between the BALF and livers of *Nlrp12^−/−^* and wild type animals. **E)** Histopathology revealed a significant amount of alveolar occlusion following *K. pneumoniae* infection in both *Nlrp12^−/−^* and wild type mice. The magnification bar at 10× = 10 µM and 40× = 5 µM. **F)** Histology scoring confirmed that no significant differences were identified between the *Nlrp12^−/−^* and wild type mice. **G)** A significant increase in TNFα, IL-6 and IL-1β was observed in the BALF from all *K. pneumoniae* challenged animals. Saline, n = 3; *Nlrp12^−/−^*, n = 13; Wild Type, n = 15. Experiments were repeated 3 separate times with a minimum of 7 *K. pneumoniae* infected *Nlrp12^−/−^* and 7 wild type mice in each group. n.s. = not significant; p = 0.0917.

The local administration of *K. pneumoniae* to the lungs results in significant morbidity, mortality, pneumonia and systemic manifestations of sepsis [1]. *Nlrp12^−/−^* mice were challenged via i.t. installation with 7.4×10^4^ CFUs of *K. pneumoniae*. Previous studies have shown that disease progression peaks at 48 hrs post-infection in wild type C57Bl/6 mice in this *K. pneumoniae* model [1]. To assess the clinical manifestations of disease progression, body temperature was utilized as a highly sensitive surrogate marker for disease progression. As shown in **Figure 3A**, 48 hrs after *K. pneumoniae* exposure, infected animals demonstrated a significant decrease in body temperature. However, no significant differences were detected between *Nlrp12^−/−^* and wild type animals (**Figure 3A**). Airway inflammation was assessed by BALF cellularity, which revealed a significant increase in total cellularity following *K. pneumoniae* infection (**Figure 3B**). Additional morphological analysis of the BALF cellularity revealed that the increase in cellularity was associated with a significant influx of neutrophils (**Figure 3C**). Notably, *Nlrp12^−/−^* mice exhibited significantly reduced levels of monocytes and lymphocytes compared to wild type mice. However, the overall significance of the reduced numbers of these specific cell populations in the *K. pneumoniae* model is unknown as global disease progression was unaffected. Local and systemic bacteria burdens can dramatically influence the host innate immune response and typically reflect disease progression. To measure the bacteria load, BALF was collected and livers were harvested from mice 48 hrs after *K. pneumoniae* infection. Similar amounts of bacteria were observed in the lungs and livers of *Nlrp12^−/−^* and wild type mice, and no significant differences in bacteria burden were observed between *Nlrp12^−/−^* and wild type animals (**Figure 3D**). This indicates that NLRP12 does not influence *K. pneumoniae* clearance or dissemination in this acute lung infection model.

*K. pneumoniae* infection is characterized by the development of severe pneumonia and parenchymal disease. Thus, to determine if histopathology was significantly altered in *Nlrp12^−/−^* mice, lungs were harvested 48 hrs post-infection and histopathology was evaluated. As expected, all of the mice challenged with *K. pneumoniae* showed significantly increased inflammatory cell infiltration and extensive alveolar space occlusion (**Figure 3E**). Subsequent histological scoring revealed that there were no significant differences in lung histopathology between *Nlrp12^−/−^* and wild type animals (**Figure 3F**). Together, these data suggest that NLRP12 deficiency does not alter the clinical progression of *K. pneumoniae* infection in mice.

In addition to inducing robust airway inflammation, *K. pneumoniae* infection also induces the local and systemic production of several cytokines that have been previously associated with NLRP12 function [1]. As shown in **Figure 3G**, airway challenges with *K. pneumoniae* induced a significant increase in BALF TNFα, IL-6 and IL-1β levels. In the case of TNFα, we consistently observed a trend towards attenuated levels in the *Nlrp12^−/−^* mice; however, the levels of TNFα were never found to be significantly different between the *Nlrp12^−/−^* and wild type animals (**Figure 3G**). Likewise, no statistically significant differences were detected in BALF IL-6 or IL-1β between the wild type and *Nlrp12^−/−^* mice (**Figure 3G**). Together, these data suggest that NL*RP12* does not play a substantial role in regulating the production of several critical cytokines in response to *K. pneumoniae* airway infection.

### NLRP12 does not Play a Role in the in vivo Host Immune Response to *Mycobacterium tuberculosis* Infection

In contrast to the acute lung infection caused by *K. pneumoniae*, *Mycobacterium tuberculosis* (*Mtb*) is a chronic lung pathogen. Previous data obtained from an *Mtb* infected human macrophage line indicates that proinflammatory cytokine levels are increased in the absence of NLRP12 [10]. These data demonstrate that NLRP12 is important for dampening the host immune response during *Mtb* infection. Thus, we tested if the absence of NLRP12 would cause an overzealous host immune response leading to increased morbidity, mortality and lung inflammation during *in vivo Mtb* infection.

**Figure 4:**
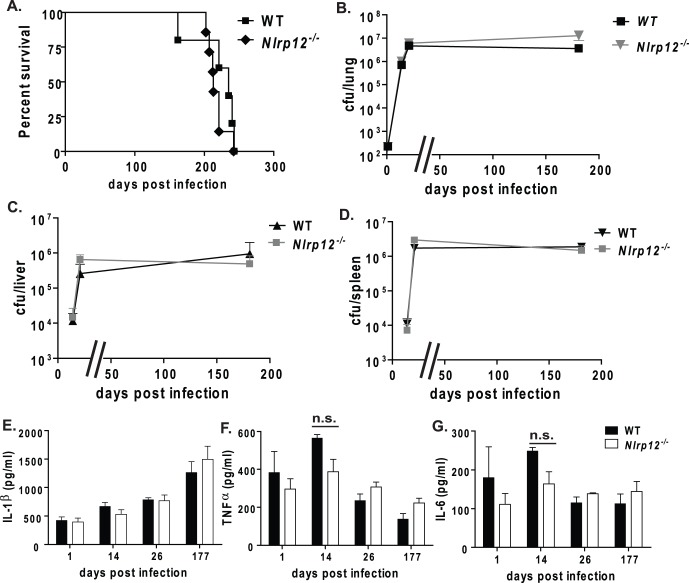
NLRP12 is not protective during *Mtb* infection. **A)** *Nlrp12* ***^−/−^*** mice infected with *Mtb* had no difference in survival compared to wild type mice (median survival was 217 days and 220 days, respectively). **B–D)** Bacterial burden in the lungs, liver, and spleen was comparable between *Nlrp12^−/−^* and wild type mice at all time points assessed. **E)** Similar amounts of IL-1β were observed in whole lung homogenates from *Nlrp12* ***^−/−^*** mice compared to wild-type lungs during both early and chronic phases of *Mtb* infection. **F–G.** Cytokine measurements of TNFα (**F**) and IL-6 (**G**) showed that *Nlrp12* ***^−/−^*** and wild-type lungs had similar amounts of cytokines at one day post-infection and during chronic *Mtb* infection. At 14 days post-infection, *Nlrp12* ***^−/−^*** lungs had reduced cytokines compared to wild-type lungs that neared significance (p = 0.056 and p = 0.06, respectively). Bacteria burden and cytokine measurements at each time point were taken from 3 mice per genotype per time point. *Nlrp12^−/−^*, n = 19; Wild type, n = 17; Experiments were repeated 2 separate times with a minimum of 12 *Mtb* infected *Nlrp12^−/−^* and wild type mice in each group. At least 3 mice per strain per time-point were used for CFU and cytokine assessments.

*Nlrp12^−/−^* and wild type mice were infected with *Mtb* via aerosol infection. We found that the mean survival was 217 days post-infection for *Nlrp12^−/−^* mice and 220 days for wild type mice (**Figure 4A**). We did not observe any differences in morbidity or mortality between the *Nlrp12^−/−^* and wild type animals, indicating that NLRP12 does not play a prominent role in host protection during *Mtb* infection. In addition to survival, we also sought to determine if NLRP12 has an effect on bacterial growth during *in vivo* infection by measuring the bacterial burden within the lungs at various time points post-infection. The lungs of *Nlrp12^−/−^* and wild type mice were harvested at 1, 14, 26 and 177 days post-infection to evaluate bacterial burden and cytokines following *Mtb* infection. We did not observe any differences in the amount of *Mtb* present within the lungs of *Nlrp12^−/−^* or wild type mice at any time point post-infection (**Figure 4B**). In addition to the local bacteria burden, we also measured the ability of *Mtb* to disseminate to secondary sites of infection. Bacterial dissemination to the liver and spleen occurs by 14 days post-infection in wild type C57Bl/6 mice [22]. We found no differences between the bacterial burden of the liver and spleen when we compared *Nlrp12^−/−^* and wild type animals (**Figure 4C–D**). These results indicate that NLRP12 does not influence *Mtb* growth or dissemination at either the primary or secondary sites of infection.

To assess the host inflammatory response to *Mtb*, the proinflammatory cytokines IL-1β, TNFα, and IL-6 were measured from lung homogenates of *Nlrp12^−/−^* and wild type mice (**Figure 4E–G**). The amount of lung IL-1β increased in both *Nlrp12^−/−^* and wild type mice as *Mtb* infection progressed. TNFα and IL-6 levels were also comparable between the lungs of *Nlrp12^−/−^* and wild type mice at one day postinfection and during the chronic phase of the infection. At day 14, we found that *Nlrp12^−/−^* lungs had reduced TNFα and IL-6 compared to wild type mice, which approached significance (p = 0.056 and p = 0.06, respectively). However, the physiological relevance of these findings is currently unclear and is in contrast to the anti-inflammatory role previously described for NLRP12 in human monocytic cell lines. This finding is reminiscent of NLRP3, which profoundly influences IL-1β production in cultured macrophages in response to *Mtb*, but does not significantly influence the *in vivo* model [22], [34]. Together, these data suggest that NLRP12 does not significantly affect lung cytokine levels, bacterial burden or mouse survival in an *in vivo* model of *Mtb* infection. Thus, we conclude that NLRP12 does not play a significant role in attenuating or exacerbating *Mtb* infection.

**Figure 5:**
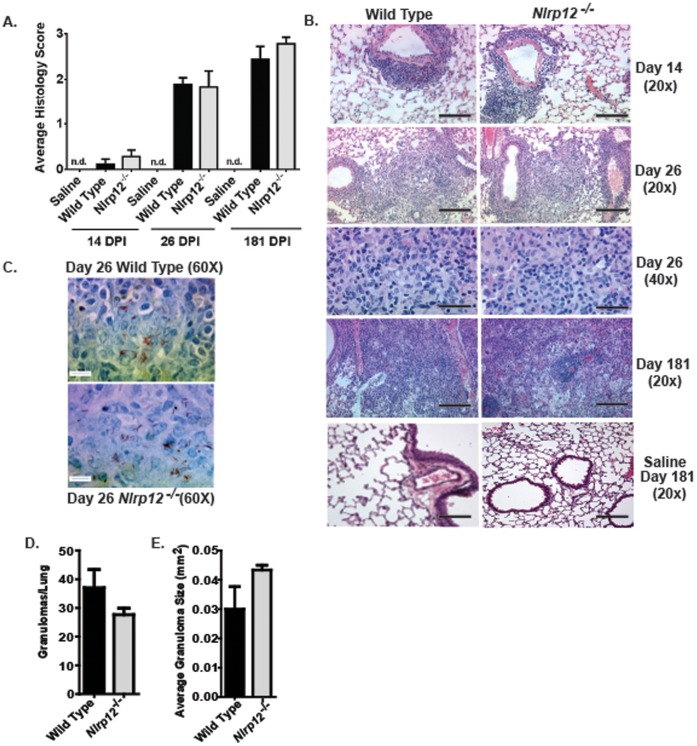
Lung histopathology and *Mtb* granuloma formation is unaltered in *Nlrp12* deficient mice. Histopathological analyses of lung sections were conducted at 14, 26 and 181 days post infection (DPI) with aerosol *Mtb*. **A)** Semiquantative histology scoring revealed a similar increase in lung inflammation over the course of the *Mtb* infection between *Nlrp12^−/−^* and wild type mice. **B)** Histopathological progression of leukocyte infiltration at 14, 26 and 181 DPI. Mild to moderate perivascular cuffing was observed around the vasculature of the lungs by 14 DPI. Extensive leukocyte infiltration around the vasculature, airways and throughout the lung parachema was observed by 26 DPI. Large, well formed granulomas were observed at 181 days post infection, which affected extensive regions of the lungs. No signs of abnormal inflammation or granuloma formation were detected in the saline treated mice at any time point evaluated. The magnification bar at 20× = 10 µM and 40× = 5 µM. **C)** Images from acid fast stained lung sections revealed that the leukocyte infiltration was associated with concentrated areas of bacteria infiltration and was consistent with early stage granuloma formation. The magnification bar at 60× = 5 µM. **D–E)** The number of granulomas per lung and the size of each granuloma was determined and no significant differences in granuloma formation was observed between either *Nlrp12^−/−^* or wild type mice. Experiments were repeated 2 separate times with a minimum of 12 *Mtb* infected *Nlrp12^−/−^* and 12 wild type mice in each group. At least 3 mice per strain per time-point were used for analysis.

Chronic *Mtb* infection results in a significant increase in lung inflammation and the formation of highly structured granulomas. A diverse array of cytokines has been shown to influence the formulation and maintenance of *Mtb* granulomas, including TNFα and IL-6 [35], [36]. These cytokines are important for the innate immune response during the initial exposure of the host to *Mtb*, but they are also important for regulating the host immune response during chronic *Mtb* infection. We observed a trending decrease in lung TNFα and IL-6 levels in the *Nlrp12^−/−^* mice. Thus, we next sought to evaluate lung histopathology over the time course of *Mtb* infection. Lungs were harvested at 14, 26 and 181 days post-infection, which represents the early growth phase, the entry to the persistence phase and the chronic phase of *Mtb* infection in C57Bl/6 mice, respectively [24]. Lung sections at each time point were evaluated and histopathological features were scored. As seen in **Figure 5A**, we observed significant changes in lung histopathology over the time course of the infection. During the early stages of the infection (Day 14), neutrophilic infiltration was concentrated around the vasculature (**Figure 5B**). However, as the disease progressed, we observed increased areas of concentrated immune cells throughout the lung parenchyma, which is indicative of granuloma lesions (**Figure 5B**). No significant differences in lung inflammation were observed between *Nlrp12^−/−^* and wild type animals. In addition to assessments of inflammation, paraffin embedded lung sections were stained with Ziehl-Neelsen acid fast stain to identify *Mtb in situ*. Our earlier data suggested that the overall lung bacterial burden was unaffected by the loss of NLRP12 (**Figure 4B**). Consistent with these earlier findings, we did not detect any differences in the bacteria burden or bacterial localization in the *Nlrp12^−/−^* and wild type mice, shown by acid-fast staining for *Mtb* bacilli (**Figure 5C**). In all cases, the majority of the bacteria were associated with the granulomas. We also characterized the granulomas in each lung section as previously described [22]. As shown in **Figure 5D–E**, the number of granulomas observed per lung and the average size of the granulomas were not significantly different between the *Nlrp12^−/−^* and wild type mice. Together, these data demonstrate that the host response to *Mtb*, including granuloma formation and maintenance, are unaffected by the loss of NLRP12.

## Discussion

Several NLRs have been shown to negatively regulate NF-κB signaling [7], [8], [9], [10], [11], [12], [13]. Of these NLRs, NLRP12 is the prototypical member and has been shown to negatively regulate components of both canonical and non-canonical NF-κB pathways *in vitro* and more recently found to display this role *in vivo* in colitis and colon cancer models [14], [16]. The canonical NF-κB signaling pathway is a critical modulator of innate immunity and is essential for host protection against pulmonary *K. pneumoniae* and *Mtb* infection [31], [37], [38]. While components of the non-canonical NF-κB and NIK signaling pathways have not been directly evaluated in the context of *K. pneumoniae* or *Mtb* infection, LPS from *Salmonella enterica* has been shown to activate both canonical and non-canonical NF-κB signaling pathways [39]. Likewise, NIK deficient *aly/aly* mice have demonstrated protection in LPS models of bone resorption and osteoclastogenesis [40]. Thus, elements of the non-canonical pathway likely influence host innate immunity to several gram negative bacteria species *in vivo*.

NLRP12 has been previously shown *in vitro* to function as a negative regulator of the host innate immune response to both LPS and *MTb* in human THP-1 monocytic cell lines [10], [12]. This function was shown to be associated with the ability of NLRP12 to interfere with both TLR2 and TLR4 pathways and antagonize signaling events downstream of MyD88, IRAK-1, TRAF2, TRAF6 and RIP1. The mechanism associated with this attenuation was attributed to the inhibition of IRAK-1 hyper-phosphyorylation [10]. While these initial studies were essential for the characterization of NLRP12, the physiological relevance of this NLR in infectious diseases is unclear. The previous studies were all conducted *in vitro* and utilized cultured human cell lines [10], [11], [12], [26]. Recent studies have begun to examine the *in vivo* role of NLRP12 utilizing *Nlrp12^−/−^* mice. In studies evaluating the development of allergic airway inflammation, no significant differences were observed between *Nlrp12^−/−^* mice and wild type animals [41]. However, NLRP12 was shown to influence the development of contact hypersensitivity and colitis associated cancer, which are both disease models with a strong innate immune system component. In the contact hypersensitivity models, dendritic cells and neutrophils from *Nlrp12^−/−^* mice failed to respond to select chemokines, which resulted in reduced migration and attenuated disease progression [15]. However, in models of experimental colitis and tumorigenesis, *Nlrp12^−/−^* mice demonstrated significantly increased inflammation and cancer progression associated with reduced inhibition of NF-κB signaling [14], [16]. Thus, it appears that NLRP12 displays distinct tissue- and disease-specific functions *in vivo*. Together, the previous *in vitro* findings and the *in vivo* data associated with the innate immune response suggested that the *Nlrp12^−/−^* mice would serve as an appropriate model to evaluate disease pathogenesis during bacterial pneumonia and *Mtb* infection. However, as our data shows, we were unable to identify a functional role for NLRP12 in either disease.

Together, these data demonstrate the importance of validating findings from cell culture/cell lines to mouse models. This is especially evident in the *Mtb* findings, which showed a convincingly strong *in vitro* phenotype for NLRP12 that was not apparent in our *in vivo* experiments. Likewise, previous *in vitro* data suggested that we would observe a significant *in vivo* phenotype in the *Nlrp12^−/−^* mice following LPS stimulation and *K. pneumoniae* infection, which we did not observe in our current study. Similar disconnects have been observed between the *in vitro* data and *in vivo* data for several NLRs and inflammasome adaptor proteins. For example, recent reports utilizing human cell lines and primary cells from knockout mice reported that components of the NLRP3 inflammasome were necessary for the host immune response against *MTb* infection [22], [42], [43]. However, follow-up studies utilizing *Nlrp3^−/−^* and *Caspase-1^−/−^* mice revealed that these inflammasome components did not directly affect the host immune response to *in vivo Mtb* infection [22], [34], [44]. Thus, many NLRs, including NLRP12, likely function through unpredictable mechanisms in the complex *in vivo* environment.

The most likely explanation for the discrepancy between the previous *in vitro* findings for NLRP12 and the data presented here, which suggest that NLRP12 does not significantly contribute to the *in vivo* host innate immune response to the pathogens evaluated, is that the studies are fundamentally different (i.e. *in vivo* vs. *in vitro*; human vs. mouse). However, it is possible that NLRP12 does contribute to the host innate immune response in a temporal, cell type-, tissue-, dose- or PAMP-specific manner and the models utilized here were too broad to discern its function. This hypothesis is supported by other findings that have shown differential and transient expression of NLRP12 in human and rodent cells [10], [15], [45]. Of particular relevance to the current work, NLRP12 was shown to be significantly elevated in cells collected from the BALF of rats that were challenged *in vivo* with either silica particles or LPS [45]. However, this up-regulation was shown to be transient, temporal and PAMP/DAMP specific [45]. The *in vivo* bacteria models utilized in the current study are associated with robust immune responses, which may obscure the contribution of indirect or subtle regulators of innate immunity. Thus, it is possible that the *in vivo* contribution of NLRP12 may have been obscured by the high levels of inflammation, which was not a factor in the previous contact hypersensitivity study [15]. In an attempt to circumvent this issue, we utilized LPS as a less robust, bacteria-free model to assess the innate immune response. However, similar to the bacteria studies, we also failed to detect a significant role for NLRP12. It is certainly possible that other *in vivo* models, which combine lower levels of inflammation with higher resolution analyses, may reveal a role for NLRP12 in mediating the innate immune response to pathogens. For example, a recent study showed a role for NLRP12 in inflammasome formation and IL-1β/IL-18 maturation in response to a subcutaneous infection with a genetically attenuated strain of *Yersinia*, but not other activators that engage the NLRP3 inflammasome [27]. Thus, it appears that the inflammasome function of NLRP12 is spatial and/or pathogen specific.

A second possible explanation for the discordance between the *in vitro* and *in vivo* findings is that other genes may be compensating for the loss of NLRP12 *in vivo*. Several recent papers have identified other NLRs that function as negative regulators of canonical and noncanonical NF-κB signaling, many of which may be capable of compensating for the loss of NLRP12. For example, NLRC5 has been shown to attenuate NF-κB signaling following *in vitro* PAMP stimulation [46], [47]. NLRC5 has also been suggested to inhibit NF-κB signaling through its interaction with IKKα and IKKβ [47], [48], although controversy exists regarding its negative regulatory function [49]. In addition to members of the NLR family, the NF-κB pathway is highly regulated, both positively and negatively, by a large assortment of other proteins. Thus, it is possible that some additional negative regulators may function, either up-stream or down-stream, to compensate for the loss of NLRP12 activity during lung inflammation.

Our data suggests that NLRP12 does not contribute to host defense in response to a limited selection of PAMPs and pathogens. However, it should be noted that future *in vivo* assessments in the lung or in other tissues using other model systems may discern novel functions for NLRP12 in mediating innate immunity. Here, we assessed the role of NLRP12 in acute and chronic models of bacterial pneumonia and *Mtb* infection. Each of these diseases impact a growing number of humans world wide and it is essential that we expand our understanding of the underlying disease mechanisms associated with the host immune response in each of these cases. The lack of a role for NLRP12 in the disease processes assessed here is encouraging from the aspect of pharmacological inhibitors that target other inflammatory disorders, such as contact hypersensitivity or inflammatory bowel disease, where NLRP12 was found to play a role in a disease model.
