# Correction: Characterization of NLRP12 during the *In Vivo* Host Immune Response to *Klebsiella pneumoniae* and *Mycobacterium tuberculosis*

**DOI:** 10.1371/annotation/2190ebe1-452c-4966-9f74-9813378524d4

**Published:** 2013-11-05

**Authors:** Irving C. Allen, Erin McElvania-TeKippe, Justin E. Wilson, John D. Lich, Janelle C. Arthur, Jonathan T. Sullivan, Miriam Braunstein, Jenny P. Y. Ting

This article was republished on November 4, 2013 due to the incorrect posting of Figure 2. Please download the article again to view the correct figures. 

